# Direct and indirect medical costs of bladder cancer in Iran

**DOI:** 10.1186/s12962-023-00416-0

**Published:** 2023-01-16

**Authors:** Mehdi Raadabadi, Rajabali Daroudi, Kazem Zendehdel, Ali Akbar Haghdoost, Mohammad Reza Ebadzadeh, Hamideh Rashidian

**Affiliations:** 1 Health Policy and Management Research Center, School of Public Health, Shahid Sadoughi University of Medical Sciences, Yazd, Iran; 2 Department of Health Management, Policy and Economics, School of Public Health, Tehran University of Medical Sciences, Tehran, Iran; 3 Cancer Research Center, Cancer Institute of Iran, Imam Khomeini Hospital, Tehran University of Medical Sciences, Keshavarz Bulvard, PoBox: 13145-158, Tehran, Iran; 4 Cancer Biology Research Center, Cancer Institute of Iran, Tehran University of Medical Sciences, Tehran, Iran; 5 HIV/STI Surveillance Research Center, and WHO Collaborating Center for HIV Surveillance, Institute for Futures Studies in Health, Kerman University of Medical Sciences, Kerman, Iran; 6 Department of Urology, Bahonar Hospital, Kerman University of Medical Sciences, Kerman, Iran

**Keywords:** Economic burden, Bladder cancer, Prevalence, Occurrence, Iran

## Abstract

**Background:**

Bladder cancer is one of the most prevalent and costly cancers in the world. Estimating the economic burden of bladder cancer is essential for allocating resources to different sectors of health systems and determining the appropriate payment mechanisms. The present study aimed at estimating the economic burden of bladder cancer in Iran.

**Methods:**

In this study, we used a prevalence-based approach for estimating the economic burden of bladder cancer. Direct and indirect costs of bladder cancer were calculated using the cost of illness and human capital approaches. Data were collected using a researcher-made checklist obtained from several sources including Iran bladder cancer clinical practice guideline, the Statistical Center of Iran, Iran’s Ministry of Cooperatives, Labor, and Social Welfare, Relative Value of Health Services (RVHS) book and Iranian Food and Drug Administration organization.

The analyses were done by Microsoft Excel 2013 and Stata 13.

**Results:**

The number of the cases of 5-year prevalence of bladder cancer in Iran was estimated as 21,807 people in 2018. The economic burden of bladder cancer in Iran was estimated at US$ 86,695,474. Indirect medical costs constituted about two-third of the economic burden of bladder cancer, and mostly related to productivity loss due to mortality. Most of the direct medical costs (29.7%) were related to the stage T2–T3 and transurethral resection of bladder (31.01%) and radical cystectomy (19.99%) procedures.

**Conclusion:**

Our results showed that the costs of bladder cancer, imposed on the healthcare system, were significant and mostly related to lost production costs. The implementation of screening and diagnostic programs can improve the survival rate and quality of life of patients and reduce the cost of lost productivity due to mortality in these patients.

## Background

Bladder cancer is one of the most prevalent cancers in both genders across the world and in males it is more than three times higher than females [1].

Urinary bladder cancer (UBC) clinically is classified to non-muscle invasive bladder cancer (NMIBC) and muscle invasive bladder cancer (MIBC) [2]. In Iran, NMIBC constitutes 68% of the bladder cancer diagnosed cases [3]. NMIBC which are restricted to the urothelial cell layers (stage Ta or Tis) or only penetrate the lamina propria (stage T1). At one end of the spectrum, low-grade NMIBC rarely progress to aggressive or metastatic tumors but as many as half will recur [4, 5]. At the other extreme, high-grade NMIBC have a high malignant potential associated with significant progression [6].

Bladder cancer is usually diagnosed at an early stage, however high progression and recurrence rate of this cancer results to a high economic burden [7]. The relative 5-year survival rate of UBC is about 97% (stage I) to 22% (stage IV) [2]. Bladder cancer is one of the most costly cancers in western countries [8] and also America [9]. Bladder cancer has the highest treatment cost per patient (from the time of diagnosis until death) [7].

Due to increasing trend of bladder cancer incidence in developing countries, we expect an increase in the economic burden of this cancer in the near future [10]. So, it is important to calculate the economic burden of cancers to provide the policy makers the awareness in allocating the resources to different sectors of health systems, also, insurance departments to determine the ways of paying the service healthcare providers [11–13]. Furthermore, such studies are used as a model of evaluating different therapeutic interventions, and they can be used as a basis for determining the priorities of medical studies [14, 15].

In a study in Iran on cancer patients, although patients with Urinary system accounted for only 2% of cases, they included 6.5% of the cancer costs [16]. Another study in Iran showed that breast, lung, blood and female reproductive organs cancers were the most costly cancers in Iran [17].

To our knowledge there is no evidence about the economic burden of bladder cancer in Iran and developing countries. We aimed to estimate the economic burden of bladder cancer in Iran for planning and policy making goals.

## Methods

In this study we used 5-year bladder cancer prevalence by stage calculated in our previous study (refrence3). In the mentioned study, we used a prevalence-based approach to calculate the economic burden of bladder cancer in Iran for 2018 by a Markov model. More details of the Markov model and estimating procedure are presented elsewhere [3]. The we estimated both direct and indirect costs of bladder cancer as follows [3]:

**(1) Direct medical costs estimation**

**Fig. 1:**
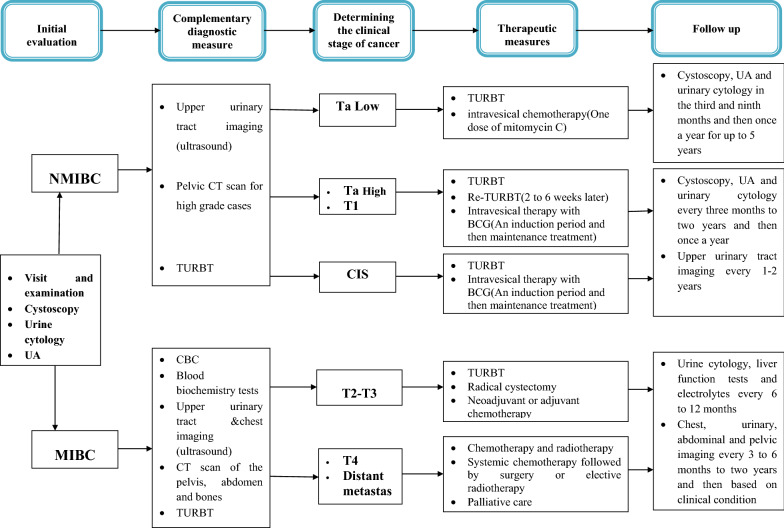
Summary of the process of diagnosis, treatment and follow-up of bladder cancer

To estimate the total direct medical costs of bladder cancer in Iran in 2018, we multiplied stage specific prevalent number of cases by the average cost per patient. We estimated the average costs per patient multiplying the frequency of health care services use per patients by the average price of the services. Health care services included diagnostic and treatment procedures and to identify the number of each services used per patient, we used Iran bladder cancer clinical practice guideline. The mentioned clinical practice guideline has been developed by cooperation of many research institutes and urology groups all over the country and published by the Ministry of Health, Treatment, and Medical Education of Iran [18]. The diagnostic and treatment services of bladder cancer by stage of the disease are depicted in Fig. 1.

We estimated the inpatient services average costs using our previous study results in 2015 [19] and updating the results for 2018 based on inflation rate of medical services in Iran. We used the Relative Value of Health Services (RVHS) book and medical tariffs approved by the government of Iran in 2018 [20] for the price of outpatient services including specialist visits, diagnostic tests, imaging, and etc. To extract the price of drugs we used the Iranian Food and Drug Administration organization data [21].

**(2) Indirect costs estimation**

Indirect costs of bladder cancer included both the morbidity and mortality cost due to bladder cancer. In this study, we used the human capital approach for estimating indirect costs [22] of bladder cancer in Iran. In this approach, we supposed the value of the lost production equal to the person’s income or wage.

*Estimation of the morbidity cost*

To estimate the Morbidity costs, we calculated the productivity loss due to bladder cancer incidence. Sometimes, patients affected by bladder cancer have to stop their daily activity for receiving healthcare. Furthermore, patient affected by high-grade cancer usually are not able to work anymore for the rest of their lifetime. This results in economic losses for both patients and the society. To estimate the productivity loss due to bladder cancer incidence, we used age-sex specific prevalent number of cases for the year 2018 in Iran multiplied by the number of daily work absence, age-sex specific employment rates, and the average daily wage.

We assumed the number of daily work absence equal to hospitalization days and the number of outpatient visit days. We extracted the average number of hospitalization days using previous studies [19] and the average number of outpatients’ visits using the bladder cancer Iranian clinical practice guidelines. We estimated all of the mentioned statistics by stage and elapsed time from diagnosis. We considered muscle invasive bladder cancer cases (local invasion and metastasis) as totally disabled [23].

We derived Age-Sex specific employment rates from Statistical Center of Iran data [24] and considered employee’s average annual income in Iran for the year 2018 as 8565 $, Based on household income and expense survey, and the Ministry of Labor and Social Affairs data [25]. we assumed the annual productivity loss value for housewives equal to the minimum annual wage in 2018 determined by the Ministry of Labor and Social Affairs (363,857,870.01 IRR). We calculated the average daily wage dividing the employees’ average annual income by the number of workdays in 2018 (290 days).

*Estimation of the mortality cost*

To estimate the mortality cost due to bladder cancer we calculated productivity loss due to premature death. To evaluate productivity loss due to bladder cancer morality in Iran for the year 2018, we multiplied the number of potential lost years by the age-sex specific employment rates, and the average annual income.

We calculated the number of potential lost years by subtracting the sex-specific age of death from the sex-specific life expectancy multiplied by the age-sex number of bladder cancer death. We estimated the age-sex number of bladder cancer death based on the results of the developed Markov model [3] and the Globocan 2018 data [26]. Calculating productivity loss cost we considered a 3% annual discount rate [27].

### Statistical analyses

We calculated the economic burden of bladder by the sum of direct and indirect medical costs of bladder cancer. To estimate the total direct medical costs of bladder cancer, we estimated the average costs per patient multiplying the frequency of health care services use per patients by the average price of the services. To estimate the Morbidity and mortality costs, we calculated the productivity loss due to bladder cancer incidence and premature death. In this study, we did the analyses by Microsoft Excel 2013 and Stata, version 13 (Stata Corp, College Station, Texas). All cost were converted to US dollar by using the average exchange rate in 2018 (USD = IR Rials 40,864) [28].

## Results

The total direct medical costs were equal to $28,657,910. The highest percentage of the costs (29.7%) was related to T2–T3 stage and the lowest one belongs to Carcinoma in situ (7.4%; Table 1). The costs of low-grade and high-grade Ta respectively constituted 18.7 and 12.8 percent of the total direct costs.

**Table 1:** The number of patients and direct medical costs of bladder cancer by stage in Iran in 2018

Stage	Number of patients			Average cost per patient ($)		Total cost ($)			Percentage of total cost
	Year of diagnosis		Sum (percentage of total)	Year of diagnosis		Year of diagnosis		Sum	
	2014–17	2018		2014–17	2018	2014–17	2018		
Low grade (Ta)	7798	2368	10,166 (46.62)	274	1362	2,134,122	3,224,377	5,358,499	18.7
High grade (Ta)	1452	592	2044 (9.37)	1087	3553	1,578,306	2,103,355	3,681,661	12.8
T1	1850	846	2696 (12.36)	1128	3552	2,086,941	3,004,793	5,091,733	17.8
Carcinoma in situ	925	423	1348 (6.18)	1173	2473	1,084,856	1,045,946	2,130,802	7.4
T2–T3	2701	1087	3788 (17.37)	1145	4978	3,093,819	5,411,229	8,505,048	29.7
Metastatic	1040	725	1765 (8.09)	1335	3450	1,388,710	2,501,455	3,890,166	13.6
Sum	15,766	6041	21,807 (100)	721	2862	11,366,754	17,291,156	28,657,910	100

Among diagnostic and treatment procedures administered for bladder cancer cases, TURBT and radical cystectomy accounts for the highest proportion of costs which constituted about 50% of the costs direct medical costs. Imaging and BCG therapy costs were the next ones (Table 2).

**Table 2:** Direct medical costs of bladder cancer in terms of diagnostic and treatment procedures in Iran (2018)

Stage	Cost ($)							
	White-light-guided TURBT (including patology, visit, drug, …)	Intravesical mitomycin C	Urologist visit	BCG therapy	Imaging & Lab services	Radical cystectomy	Chemotherapy& radiotherapy	Total
Ta low	2,556,120	154,564	134,128	0.00	1,027,200	0.00	0.00	3,872,013
Ta High	1,278,060	0.00	44,106	1,090,376.86	422,090	0.00	0.00	2,834,633
T1	1,825,800	0.00	57,185	1,479,149.19	549,481	0.00	0.00	3,911,615
CIS	456,450	0.00	27,290	722,494.61	263,628	0.00	0.00	1,469,863
NMIBC recurrent	2,246,918	119,690	72,007	1,117,765.53	618,192	0.00	0.00	4,174,573
T2–T3	418,141	0.00	88,783	0.00	1,144,568	5,728,305	1,125,251	8,505,048
Metastases	105,407	0.00	59,837	0.00	820,590	0.00	2,904,332	3,890,166
Total	8,886,896	274,254	483,337	4,409,786.19	4,845,749	5,728,305	4,029,583	28,657,910
Percent of total	31.01	0.96	1.69	15.39	16.91	19.99	14.06	100.00

The total number of lost workdays due to bladder cancer morbidity in 2018 was equal to 1,681,931 days. About 83% of the workdays were lost by male employees. The lowest and the highest number of lost workdays were respectively related to the age groups of 40–44 (2.4%) and above 75 years (24.1%). The total cost morbidity productivity loss due to bladder cancer was equal 17,599,403 $ in Iran for the year 2018; about 96% of this value was related to men. Although the highest number of the lost workdays was related to the age group above 75 years, the cost of productivity loss was zero as a result of the zero employment rates in this group. The highest cost of morbidity productivity loss due to bladder cancer was related to the age group of 60–64 years (21.5% of the total morbidity cost; Table 3).

**Table 3:** The number of lost workdays and morbidity cost due to bladder cancer in Iran (2018)

Age	Number of lost workdays in 2018				Morbidity cost (USD)			
	Male	Female	Total	Percent of total	Male	Female	Total	Percent of total
15–39	30,966	22,039	53,005	3.2	745,228	122,528	867,756	4.9
40–44	33,756	6416	40,172	2.4	875,759	35,655	911,389	5.2
45–49	72,254	9206	81,460	4.8	1,796,912	44,220	1,841,156	10.5
50–54	135,860	16,738	152,599	9.1	2,943,324	62,598	3,005,947	17.1
55–59	184,959	25,945	210,904	12.5	3,360,537	81,490	3,442,027	19.6
60–64	223,737	38,498	262,235	15.6	3,658,575	123,483	3,782,058	21.5
65–69	216,483	49,936	266,420	15.8	2,354,468	110,660	2,465,128	14.0
70–74	169,895	40,172	210,067	12.5	1,254,429	29,513	1,283,942	7.3
75 +	332,257	72,812	405,070	24.1	0.00	0.00	0.00	0.0
Total	1,400,168	281,763	1,681,931	100.0	16,989,233	610,170	17,599,403	100

In 2018, 2383 cases of death due to bladder cancer have been estimated in Iran. The lowest and the highest number of deaths were respectively reported for the age groups of 40–44 years (28 cases) and above 75 years (1202 cases). The cost of productivity loss due to bladder cancer mortality was estimated 40,438,160.73 $. The highest percent of this cost were respectively related to the age groups of 50–54 years (19.5%) and 55–59 years (19.4%) (Table 4).

**Table 4:** The number of premature deaths and mortality cost due to bladder cancer in Iran (2018)

Age	Number of deaths in 2018				Mortality cost (USD)			
	Male	Female	Total	Percent of total	Male	Female	Total	Percent of total
15–39	21	9	30	1.3	3,271,119	313,699	3,584,818	8.9
40–4	25	4	28	1.2	2,839,321	81,955	2,921,275	7.2
45–49	59	4	63	2.6	5,534,896	64,189	5,599,109	13.8
50–54	106	13	120	5.0	7,719,827	170,492	7,890,319	19.5
55–59	144	23	167	7.0	7,628,671	215,618	7,844,288	19.4
60–64	197	27	224	9.4	6,788,518	162,025	6,950,568	17.2
65–69	235	55	291	12.2	4,213,929	149,276	4,363,205	10.8
70–74	207	51	258	10.8	1,253,671	30,883	1,284,554	3.2
75 +	908	294	1,202	50.4	0.00	0.00	0.00	0
Total	1,903	480	2,383	100.0	39,249,976	1,188,185.20	40,438,161	100

The total economic burden of bladder cancer in Iran for the year 2018 was estimated $86,695,473.86. Mortality cost due to bladder cancer was responsible for the majority part of economic burden of bladder cancer (46.6%) and only 33% of the economic burden of bladder cancer was related to direct medical costs (Table 5).

**Table 5:** Direct and indirect medical cost of bladder cancer in Iran (2018)

Type of costs	Amount (USD)	Percent of total
Direct medical costs	28,657,910	33.1
Indirect medical costs		
Morbidity cost	17,599,403	20.3
Mortality cost	40,438,161	46.6
Total	86,695,474	100.0

## Discussion

In this study, economic burden of bladder cancer in Iran (2018) was estimated US$86,695,474 based on prevalence based approach. Indirect medical cost was responsible for about 67% of the total economic burden of bladder cancer and cost due to mortality constitutes about two third of it. Among direct medical costs, T2–T3 stage had the highest proportion (About 30%) and the CIS stage had the lowest cost (7.4%). Transurethral resection of bladder tumor (TURBT) was responsible for one third of direct medical cost of bladder cancer. Males between the ages 50–64 were responsible for the majority cost of bladder cancer.

The Average direct medical costs of bladder cancer in Iran in 2018 were $1314 per prevalent case. In European Union, the annual health care expenditures due to bladder cancer accounted €6942 per prevalent case [8]. In the United States, inpatient and outpatient costs of bladder cancer were estimated at about US$7806 per prevalent case in 2010 [29]. As a result, some researchers have questioned the effectiveness of higher US spending and suggested it is driven by higher costs, unnecessary testing, and unproven medical procedures [30]. Differences in health care expenditures due to bladder cancer could be explained by health system configuration (eg, the number of hospitalization days due to bladder cancer, differences in the introduction and administration of new drugs, differences in the prices paid for the same drugs, differences in the types of medication consumed, and variations in clinical practice).

According to the stages of the disease, 56.7% of the costs were related to NMIBC and the remaining to MIBC. This finding is inconsistent with the results of the studies in America [31–33]. The findings of the study by Cooksley et al. suggested that the annual treatment cost for patients with muscle invasive bladder cancer was 70% more than the treatment cost of patients with non-muscle invasive bladder cancer [32]. In another study conducted by Avritscher et al. in America, the average cost primary treatment of bladder cancer was $6548 in non-muscle invasive stage and $42,035 in muscle invasive stage [31]. Considerable variation in the management of bladder cancer by different providers may contribute to heterogeneity of spending patterns. A variety of treatment algorithms are available for NMIBC management, and providers exert their best clinical judgment when choosing how strictly to adhere to these strategies.

In addition, the difference in results can be related to the prevalence in different countries. Regarding the higher prevalence of NMIBC than MIBC and high recurrence and progression rate is in this group that requires long term cystoscopy control and repeated interventions, the costs of NMIBC are considered higher than MIBC [2]. Furthermore, studies have shown that the care of patients with NMIBC varies significantly depending on the geographic region [34] and physician [35]. Also, the costs of caring for patients vary depending on the stages of disease; so that, these costs will increase by almost two times in the first 2 years after diagnosis [34].

According to our estimation of direct medical costs, the highest cost was related to TURBT and radical cystectomy. TURBT is the initial treatment of non-muscle invasive bladder cancer and it constitutes a major part of the total treatment costs [2]. The average cost of TURBT (including the costs of surgeon, anesthesia, operating room, hospitalization and laboratory tests) in Iran was US$1471 in 2018. The average cost of this procedure has been reported as US$4349 in America and US$2000–2500 in European countries [2].

Bacillus Calmette-Guerin (BCG) is suggested for patients with bladder cancer in CIS stage, high-grade Ta, and T1 [35–38]. According to the Iranian clinical practice guideline of bladder cancer, a 6-week period of induction therapy by BCG is suggested for patients in CIS stage, high-grade Ta, and T1 accompanying by maintenance intravesical treatment by BCG [35]. The average cost of a 6-week period of BCG injection (including the costs of the medicine and injection) in Iran (2018) has been estimated as US$4,409,786.19. The results of economic assessments suggested the intravesical immunotherapy by BCG to be cost-effective and its cost for one life-year saved is estimated to be about US$5000 [39–41].

Intravesical therapy is another treatment of bladder cancer NMIBC cases that includes immunotherapy and chemotherapy. Currently, clinical practice guidelines suggest intravesical chemotherapy to be performed immediately after TURBT for new diagnosed or recurred low-grade non-muscle invasive bladder cancer patients. Intravesical chemotherapy is usually done by using a dosage of mitomycin C 24 h after TURBT surgery [35–37]. The average cost of intravesical injection of a dosage of mitomycin C (40 mg) in Iran (2018) has been estimated US$45.4. The equivalent costs of this injection in America and England have been respectively estimated as US$219 and US$87 [2]. Although clinical practice guidelines suggest intravesical chemotherapy and economic studies have approved the cost-effectiveness of this treatment, it is not widely used even in America [42, 43].

In muscle invasive bladder cancer, based on the progress of disease and the patient’s conditions, a combination of treatments including TURBT surgery, radical cystectomy, neo-adjuvant chemotherapy, radiotherapy, and chemotherapy may be used for patients. According to suggestions of clinical practice guideline, the standard treatment of local muscle invasive bladder cancer (T2 or T3) is radical cystectomy accompanied by neo-adjuvant chemotherapy. Also, chemotherapy with or without radiotherapy has been suggested for patients with metastatic bladder cancer [35–37]. According to the data collected in this study, the average cost of cystectomy was US$948 in 2018. The costs of radical cystectomy in America and England were respectively equal to US$ 23,451 and US$ 8,090 [2]. The most prevalent bladder cancer chemotherapy drugs include Dose-Dense MVAC (methotrexate, vinblastine, doxorubicin, and cisplatin) and Gemcitabine and Cisplatin (GC) [2, 36]. As there is a significant difference between the prices of generic drugs and brand drugs, the costs of these chemotherapy drugs depend on using generic or brand drugs.

According to the findings of this study about 67% of the economic burden of bladder cancer in Iran results from the productivity loss cost. In a study conducted by Leal et al. aimed at estimating the economic burden of bladder cancer in European countries (2012), the total economic burden of bladder cancer in European countries was 4.9 billion Euros; 41% of this rate resulted from the lost production costs [8]. In a study conducted by Gerace et al. in Italy (2017), the total economic burden of bladder cancer was estimated 1.2 billion Euros; 44% of this rate resulted from the lost production costs [44]. Although productivity loss costs constitute a significant part of the economic burden of diseases, these costs are sometimes overlooked by policy makers and planners.

Since bladder cancer is more prevalent among the elderly [45], it is expected that its occurrence, prevalence, and cost increase with the population aging in the future years. According to estimations of Globocan 2018, due to demographic changes, the number of bladder cancer cases in Iran will increase by about 1.5–2 times in 2025 compared with 2018. Since the costs of diagnosis, treatment, and follow up of bladder cancer in early stages are significantly less than the progressed stages, early diagnosis and careful follow up and also, early detection of recurrence cases can decrease the costs of the patients’ treatment and follow up.

### Strengths and constraints of the study

This research is the first study to investigate the economic burden of bladder cancer in Iran. One of the strengths of this study is the use of modeling for estimation of prevalence cases in terms of stage and the occurrence year.

Although this study has investigated a major part of the economic burden of bladder cancer in Iran, however, some of the costs have not been estimated due to lack of reliable data including direct nonmedical costs (the costs of travelling and accommodation in other cities for receiving treatment; the costs of information cares and treatments); intangible costs (decreased quality of life); the costs of relieving cares and the treatments provided in the late stages of bladder cancer patients’ life (hospitalization, receiving care in ICU wards, etc.); and hidden cost such as informal payment.

Furthermore, this study has investigated the economic burden of the 5-year prevalence cases bladder cancer in Iran; whereas, some patients may report the recurrence of disease after the 5th year and they may need to receive follow up. So, this disease can impose costs on patients after the 5th year. Therefore, in this study, the economic burden of bladder cancer estimations could be less than the real value.

The other constraint of this study is estimation of direct medical costs based on some assumptions of using treatment methods. As stated previously, we assumed that in Iran, all bladder cancer patients are treated in accordance with the clinical practice guideline published by the Ministry of Health, Treatment, and Medical Education. However, although some treatments are suggested by clinical practice guidelines, they may not be used by all physicians. In this situation, the real costs of the disease will be different from the costs estimated in this study.

## Conclusion

Our results showed that the costs of bladder cancer, imposed on the healthcare system, were significant and mostly related to lost production costs and in early stages, medical costs are significantly lower than the costs of progressed stages. Considering the high cost of lost productivity due to mortality, the implementation of screening and diagnostic programs can improve the survival rate and quality of life of patients and reduce the cost of lost productivity due to mortality in these patients. Also, the results of this research can be used in economic evaluation studies to select interventions as well as preventive programs such as cancer awareness and screening programs.

## Data Availability

The data-sets used and/or analyzed during the current study available from the corresponding author on reasonable request.

## Abbreviations

UBC: Urinary bladder cancer
NMIBC: Non-muscle invasive bladder cancer
MIBC: Muscle invasive bladder cancer
CIS: Carcinoma in situ
